# Dental complications as a potential indicator of Redondovirus infection: a cross-sectional study

**DOI:** 10.1186/s12879-024-09523-6

**Published:** 2024-07-05

**Authors:** Alireza Mohebbi, Seyed Jalal Kiani, Khadijeh Khanaliha, Tahereh Donyavi, Nikoo Emtiazi, Kimia Sharifian, Maryam Mohebbi, Amytis Gholami, Farzane Behnezhad, Mohammad Abbasi-Kolli, Farzaneh Dehghani-Dehej, Farah Bokharaei-Salim

**Affiliations:** 1 Department of Virology, School of Medicine, Iran University of Medical Sciences, Tehran, Iran; 2 Vista Aria Rena Gene, Inc, Gorgan, Golestan Province Iran; 3 Research Center of Pediatric Infectious Diseases, Institute of Immunology and Infectious Diseases, Iran University of Medical Sciences, Tehran, Iran; 4 Department of Medical Biotechnology, Faculty of Allied Medicine, Iran University of Medical Sciences, Tehran, Iran; 5 Department of Pathology, Iran University of Medical Sciences, Tehran, Iran

**Keywords:** Redondovirus, SARS-CoV-2, Periodontitis, Oral health, Respiratory infection

## Abstract

**Background:**

*Redondoviridae* is a newly discovered virus family linked to oral and respiratory conditions in people, while there is still debate about whether it is also coinfected with other respiratory viruses. This study aimed to determine the frequency of Redondovirus (ReDoV) in nasopharyngeal samples and to investigate any possible links to SARS-CoV-2 infections.

**Methods:**

A polymerase chain reaction (PCR) test was conducted on 731 nasopharyngeal samples from individuals referred to medical centers in Tehran, Iran, for SARS-CoV-2 testing to investigate the prevalence of ReDoV. An oral interview was performed to complete information on dental issues and the individuals’ demographics, symptoms, and vaccination history.

**Results:**

The prevalence of ReDoV was 25.99%, and 15.26% had a coinfection with SARS-CoV-2. No notable correlation was found regarding ReDoVs and SARS-CoV-2 infections (*p* > 0.05). Women had a higher ReDoV positivity rate of 18.47% compared to men at 7.52% (*p* = 0.12), and there was no significant correlation between age groups and ReDoV presence. Nonetheless, a significant association was noted between ReDoVs and dental/gum issues (*p* < 0.0001, OR: 13.0326). A phylogenetic analysis showed that ReDoVs originated from various human-related clusters.

**Conclusions:**

These results highlight the potential for detecting ReDoVs in nasopharyngeal samples of people with gum or dental issues. Additionally, conducting more ReDoV epidemiological research and proposing oral health as a possible marker for ReDoV infections is important.

**Supplementary Information:**

The online version contains supplementary material available at 10.1186/s12879-024-09523-6.

## Background

Circular Rep-encoding single-stranded DNA (CRESS DNA) viruses are now considered ubiquitous and have been discovered on all continents, infecting various animals, including insects, birds, rodents, bats, chimps, and humans, as well as in diverse environmental and animal tissue samples [1, 2]. Recently discovered members of *Redondovirae* in a metagenomic study, a member of *Recrevirales* order [3], has been suggested as a comorbidity in human respiratory diseases [4–7]. Redondoviruses (ReDoVs) share some features with other CRESS DNA viruses but also display unique characteristics. The genomes contain ambisense open reading frames (ORFs), including a replication initiation protein (Rep) and capsid protein (Cp). They also have a third distinct ORF (ORF3) [5]. Recombination is a major mechanism contributing to Redondovirus genomic diversity [8]. This implies the high potential for emerging novel ReDoV variants in infected hosts. *Redondovirus* (ReDoV) was initially identified in bronchoalveolar lavage (BAL) samples of lung transplant patients [5], and the eukaryotic commensal amoeba *Entamoeba gingivalis* (*E. gingivalis)* was identified as the primary host for ReDoV replication [9]. ReDoVs are commonly found in the oral cavity and respiratory tract of humans, infrequently in the gut, but absent in blood, vaginal, and skin [10]. The genome of ReDoV is a circular DNA ~ 3000 nucleotides in length. Two ReDoV species, *Vientovirus* and *Brisavirus*, despite sharing 50% nucleotide similarity, belong to the *Torbevirus* genus.

ReDoV is proposed as the second most prevalent eukaryotic virus in human respiratory samples [8, 11]. Abbas et al. found ReDoV in human samples from the oral cavity (3.8%), lung (3.3%), nasopharynx (0.95%), and gut (0.59%) [5]. There is a limited understanding of ReDoV’s epidemiology, biological properties, and pathogenic potential. Abbas et al. observed higher ReDoV prevalence in critically diseased patients, suggesting a potential link to clinically relevant disorders. Accordingly, Spezia et al. aimed to investigate ReDoV DNA prevalence, distribution, and clinical consequences in 543 biological specimens, including respiratory, blood, stool, and liquor samples [11], and detected ReDoV DNA in 4% of the overall population, with the highest prevalence (11%) in respiratory tract samples. For establishing the association of ReDonVs with human-related respiratory diseases, they observed most virus-positive patients had severe respiratory diseases, and a majority had no other common respiratory viruses or microbial agents detected. However, due to the lack of definitive evidence linking ReDoV to severe respiratory tract diseases, the authors suggested further exploration of ReDoV’s potential pulmonary/extrapulmonary presence of the virus, particularly in stool samples [11]. Furthermore, da Costa et al. reported a 74.6% prevalence of ReDoVs (*Brisavirus* in 67.9% and *Vientovirus* in 64.3% of patients) in the saliva samples of COVID-19 patients vs. 50% in controls [12]. Partly, due to the small sample size and lack of evaluation on severe COVID-19 cases, this study did not find evidence for ReDoV’s influence on the course of SARS-CoV-2 infection.

The role of microbiota in COVID-19 has gained much importance, not only in the respiratory system but also in the gut and oral microbiota. Understanding the interactions of the microbiota could have implications for the diagnosis, treatment, and prognosis of SARS-CoV-2 [13]. Global distribution of ReDonVs is noted, with prevalence rates ranging from 2 to 82% in the oro-respiratory tract in healthy and diseased individuals. Spezia et al. investigated the presence and loads of ReDoV in saliva samples from 326 SARS-CoV-2-negative healthy individuals and 122 hospitalized-positive subjects [14]. ReDoV DNA was found in 61% of the 377 tested saliva samples, with a significant difference between SARS-CoV-2-negative and -positive groups [14], suggesting ReDoV infection to be primarily restricted to the respiratory tract. Due to the evidence supporting ReDoVs in the oral cavity, investigating the viral infection is crucial in oral health programs. Accordingly, periodontitis is a prevalent oral disease characterized by inflammation, destruction of tooth attachment apparatus, and alveolar bone resorption. Zhang et al. detected a significantly higher prevalence of ReDoVs in chronic periodontitis patients (71.67%) compared to healthy individuals (51.67%) [15], and confirmed a significant association between chronic periodontitis and ReDoVs infection (OR of 2.53). Also, the discovery of five novel CRESS (HPeCVs) in gingival tissue biopsies of chronic periodontitis patients, with conserved motifs and structures like that identified in the ReDoVs’ genomes [15] supports the importance of investigating oral infection with ReDoVs.

Further studies have also contributed to investigating the ReDoV association with human respiratory diseases. A recent study by Makoa-Meng et al. identified a highly correlated (93%) distribution of ReDoV with *E. gingivalis* in oral samples [16]. This finding suggests a possible interaction of ReDonVs with human-associated pathogens in the oral cavity [17]. This is also supported by the finding of three new CRESS virus families, *Naryaviridae*, *Nenyaviridae*, and *Vilyaviridae*, infect Entamoeba and Giardia in human clinical samples [18]. The finding emphasizes the importance of understanding virus-host relationships for medical implications. Further implication of potential pathogenic association of ReDoVs with respiratory tract diseases is shown in a metagenomic analysis of the sputum sample from a Spanish man with symptoms included distempered sensation, shivering, headache, respiratory distress, respiratory insufficiency, hypertransaminasemia, and thrombocytopenia after traveling to Nepal [19]. In this study, the authors identified two variants of a novel human respiratory circovirus-like virus (HRCLV) - HRCLV-HULP1 and HRCLV-HULP2, and the presence of a third ORF encoding a leucine-rich protein, a signature of Vientoviruses [19, 20]. It has also been shown that circular RNAs (circRNAs) are dysregulated in periodontitis patients with ReDoV infection [21]. This finding enhances the understanding of the mechanism underlying ReDoV-related periodontitis and provides insights that could potentially be applied in developing diagnostic markers and therapeutic strategies. Also, this could contribute to future studies of ReDoV’s contribution to other human respiratory infections like COVID-19.

Detection of the ReDoVs in various human samples, such as the respiratory tract, is essential, particularly during a SARS-CoV-2 infection. Detection of ReDoVs in the saliva of individuals with COVID-19 and pointing out a possible connection between ReDoVs and respiratory illnesses, emphasizing the importance of studying how they interact with the host’s microbiota in association with COVID-19. This study aims to explore the co-infection of ReDoVs and SARS-CoV-2 in Iran and their distribution and potential impact on diagnosis, treatment, and prognosis, providing important information on the broader significance of ReDoVs in human health. As an important finding, the association of oral health problems with ReDoV infection was addressed.

## Methods

### Study populations and data collection

In this cross-sectional study, nasopharyngeal samples from individuals referred for the molecular diagnosis of COVID-19 to one of the health centers or hospitals affiliated with Iran University of Medical Sciences (IUMS), Tehran, Iran, were included. The subjects included were well-preserved nasopharyngeal samples in viral transport media (VTM), comprised of 731 nasopharyngeal swaps from March 2023 to August 2023. Demographical information, including age and sex, as well as the Real-Time polymerase chain reaction (RT-PCR) results of COVID-19, were obtained from the medical centers’ databases as reported before [22]. Referrals who tested negative for SARS-CoV-2 and did not report any respiratory were included to compare the prevalence of ReDoV in the studied population.

Further information was collected through a questionnaire and an interview with the referrals. The questionnaire included questions about the COVID-19 vaccine details (type and number of doses), influenza vaccine status, underlying diseases, drug usage, dental issues, and clinical symptoms. In this regard, individuals were informed about the study in which their biological samples were used and asked to complete the questionnaire (see Additional file 1).

### Viral genome extraction and amplification of the ReDoV genome using PCR

The preserved in a viral transport media (VTM) were retrieved from ˗80 °C freezer before viral DNA extraction. The viral DNA was extracted from the oropharyngeal and nasopharyngeal samples using the QIAamp® DNA Mini kit (Qiagen GmbH, Germany) in accordance with the manufacturer’s protocols. Then, the quantity and quality of the isolated DNA and RNA were determined using a NanoDrop spectrophotometer instrument (Thermo Fisher Scientific, Wilmington, MA).

The DNA of ReDoV was amplified by PCR method using the following primers: Redondo-Forward, 5’- ATAGTACCATCAGAAACAGGTG- 3’ (nucleotides 1709–1730) and Redondo-Reverse, 5’- GTTTCACAAGTGACAACGAAA- 3’ (nucleotides 1895–1875). The PCR assay was carried out in a 25 µL mixture reaction containing 2.5 µl of 10X Ex Taq buffer (Mg2 + free), 2.0 µl dNTPs Mixture (25 mM each), 2.0 µl of MgCl2 (25 mM), 10 pmol of each primer, and 1.0 U of Ex Taq DNA polymerase (TaKaRa Biotechnology [Dalian] Co., Ltd., Shiga, Japan). In addition, 5 µl of extracted viral DNA was added to the mixture as a template. The PCR conditions were as follows: initial denaturation at 95 °C for 10 min, 35 cycles of 30 s at 95 °C, 30 s at 56 °C and 35 s at 72 °C, followed by a final extension of 5 min at 72 °C.

A PCR efficiency was also performed by making a serially diluted (1:10) DNA sample of a strongly positive sample. Accordingly, a relative qPCR was performed with the same ReDoVs pair of primers and the same amounts of ingredients using the Rotor-Gene Q instrument (QIAGEN, Germany). Linearity of the diluted sample and cycle threshold (Ct) was reported as previously demonstrated [23].

### DNA sequencing and phylogenetic analysis

15 ReDoVs positive samples were randomly selected for DNA sequencing. In this regard, after amplification by PCR and purification with a highly pure PCR product purification kit (Roche Diagnostic, Mannheim, Germany), samples were sequenced bi-directionally using the dye termination method by the ABI 3730 XL sequencer.

Different oral-, lung-, and human respiratory-associated, as well as unassigned ReDoVs’ sequences, were retrieved from NCBI for multiple sequence alignment (MSA) with the sequenced genome from this study. The sequencing results were MAFFT v7 [24, 25]. Accordingly, MSA was performed with the G-INS-1 strategy to establish an accurate guide tree as described before [26]. The statistical significance of the phylogenetic tree was evaluated using the bootstrap method (1,000 replicates). A closely related *Anellovirus* genome was also used as the outgroup to root the tree. A Newick graph-theoretical trees file format was saved and used to reconstruct the radial phylogram using the Interactive Tree Of Life (iTOL; https://itol.embl.de/) online tool [27].

### Statistical analyzes

Statistical analysis was performed by MS-Excel XLSTAT v2019 software package, as reported before [28, 29]. The normality of the quantitative variable (age) was tested using the Kolmogorov-Smirnov test and was analyzed by the Kruskal–Wallis test. The statistical differences between categorical variables were assessed using the Contingency table, and the strength of the test was approved by Fisher’s exact test. A *p*-value ≤ 0.05 with an alpha value of 0.05 and a 95% confidence interval (CI 95%) was considered statistically significant.

### Ethical approval

Ethical approval for the present study was obtained from the ethics committee of IUMS, Tehran, Iran (ethical code: IR.IUMS.FMD.REC.1402.351), in accordance with the Second Declaration of Helsinki. All the interviewed people were informed about this survey, and a written consent form was obtained from all participants before their enrolment.

## Results

### Demographic characteristics of the study

To investigate the association of ReDoV with human-associated viral diseases, the prevalence of ReDoV among individuals undergoing SARS-CoV-2 diagnostic testing was analyzed. The subjects consisted of 486 (66.48%) females (minimum age of 4 years and a maximum age of 83 years) and 245 (33.52%) males (ranging from 9 years to 99 years old) with a mean age of 37.97 ± 13.36 and 44.94 ± 17.06, respectively. For identifying any age-specific patterns, differences in susceptibility, or trends that might be present in different age groups and the ease of interpreting the results and understanding the prevalence of ReDoVs across different age groups, the subjects were divided into seven different age categories. According to the recorded documents of the subjects, the age distribution of this study illustrated a predominant representation in the 30–39 age group (29.82%), followed by individuals aged 40–49 (23.39%) and 20–29 (19.97%) (See Table 1).

**Table 1:** Demographic and clinical characteristics. Data were obtained either through the medical center records or by interviews with participating individuals

Variable\Statistic	Categories	Frequency per category ^¥^	Rel. frequency per category (%)
**Age category**	1–9	3.00	0.41
	10–19	32.00	4.38
	20–29	146.00	19.97
	30–39	218.00	29.82
	40–49	171.00	23.39
	50–65	93.00	12.72
	> 65	68.00	9.30
**SARS-CoV-2 result**	Negative	600.00	82.08
	Positive	131.00	17.92
**ReDoV results**	Negative	541.00	74.01
	Positive	190.00	25.99
**Coinfection**	SARS-CoV-2/ReDoVs ++	28.00	3.83
**COVID-19 vaccine**	No	21.00	2.87
	Yes	710.00	97.13
**Vaccination frequency**	Five times	4.00	0.55
	Four times	107.00	14.64
	No	21.00	2.87
	One time	22.00	3.01
	Three times	334.00	45.69
	Two times	243.00	33.24
**Type of vaccine**	None/did not remember	514.00	70.31
	AstraZeneca	19.00	2.60
	Sinopharm	145.00	19.83
	Sputnik	5.00	0.68
	Others	43.00	5.88
**Influenza vaccination**	No	680.00	93.02
	Yes	51.00	6.98
**Underlying disease**	No	623.00	85.23
	Yes	108.00	14.77
**Taking medication**	Not remembered	2.00	0.27
	No	625.00	85.50
	Yes	104.00	14.23
**Gum/dental issues**	No	534.00	73.05
	Yes	197.00	26.95
**Fever**	No	523.00	71.55
	Yes	208.00	28.45
**Headache**	No	530.00	72.50
	Yes	201.00	27.50
**Confusion**	No	663.00	90.70
	Yes	68.00	9.30
**Chills**	No	602.00	82.35
	Yes	129.00	17.65
**Bone ache**	No	572.00	78.24
	Yes	159.00	21.75
**Dry cough**	No	581.00	79.48
	Yes	150.00	20.52
**Sputum cough**	No	656.00	89.74
	Yes	75.00	10.26
**Chest pain**	No	680.00	93.02
	Yes	51.00	6.98
**Shortness of breath**	No	672.00	91.93
	Yes	59.00	8.07
**Runny nose**	No	621.00	84.95
	Yes	110.00	15.05
**Stuffy of nose**	No	657.00	89.88
	Yes	74.00	10.12
**Stomach bleeding**	No	729.00	99.73
	Yes	2.00	0.27
**Deceased Smell**	No	658.00	90.01
	Yes	73.00	9.99
**Deceased taste**	No	684.00	93.57
	Yes	47.00	6.43

^**¥**^ Sum of observation in each category

### Prevalence of SARS-CoV-2 among participants

The RT-PCR results of SARS-CoV-2 were obtained from the referrals’ records. As shown in Tables 1 and 131/731 (17.92%) were positive for SARS-CoV-2. Regarding vaccination status, a vast majority (97.13%) had received the COVID-19 vaccine, with the most common frequency being three times (45.69%). Sinopharm was the most prevalent vaccine type (19.84%) among those surveyed. Further information obtained by interviewing participants included gum or dental problems during their referral to the medical centers, clinical symptoms associated with COVID-19 disease (smelling or testing senses, fever, headache, bone ache, cough, chest pain, shortness of breath, and other flu-like symptoms, as well as underlying diseases and the type of medication (data are not shown), and influenza/COVID-19 vaccination data were. The presence of underlying diseases was noted in 14.77% of the population, while 14.23% reported taking medication. The underlying diseases included hypertension, diabetes, nephrotic syndrome, anemia, heart problems, arthritis rheumatoid (AR), asthma, stomach reflex, autism, blood fat, migraine, bronchitis, cancer, chemical veteran, endocarditis, fatty liver, gallstone, HIV, autoimmune disease, lung disease, allergy, hyperlipidemia, hyperprolactinemia, lupus, seizures, hyperthyroidism, hypothyroidism, intestinal bleeding, intestinal ulcer, Behçet’s disease, lymphoma, multiple sclerosis (MS), nervous and mental problems, osteoporosis, rheumatism, prostate cancer, myopathy, and psoriasis. To investigate the ReDoV genome further, the nasopharyngeal samples of the consent subjects were retrieved from the − 80 °C refrigerators, and viral genome extraction was performed, as described in the materials and methods section.

**Fig. 1:**
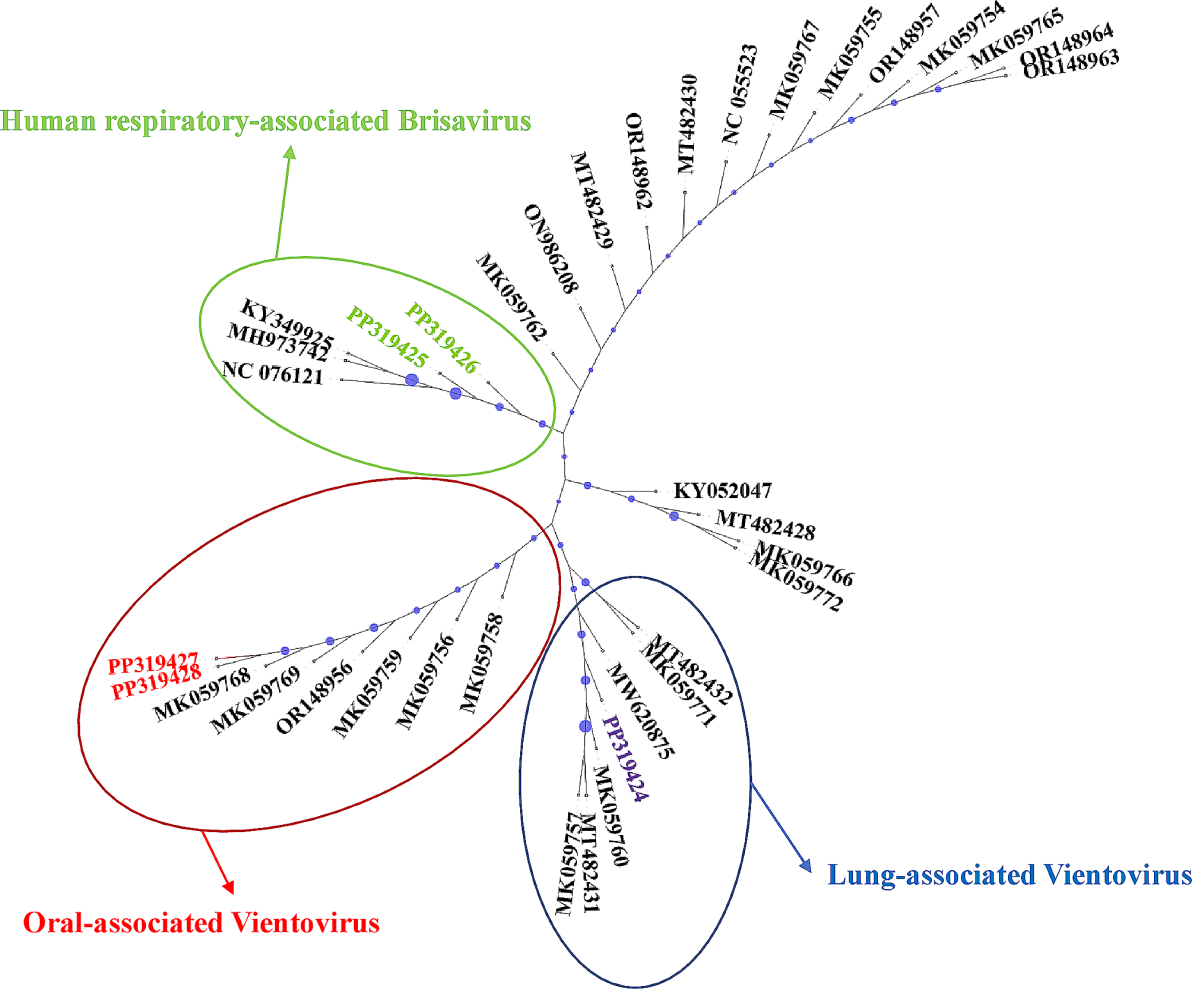
Radial phylogram re-rooted with Mosquito VEM Anellovirus SDBVL A (NC_076121.1). The sequenced data of ReDoVs from the present study are colored. PP319426 and PP319425 are phylogenetically related to the human respiratory-associated Brisaviruses. PP319427 and PP319428 are genetically close to oral-associated Vientoviruses, and PP319424 is related to lung-associated Vientoviruses. Blue circles represent bootstraps ranging from 4 (smallest) to 100 (largest)

The ReDoV sequences obtained from the amplified samples were submitted to GenBank, and they will be available with accession numbers PP319424 to PP319438. A phylogenetic analysis was performed to measure further the genetic distance of the sequenced data with already known ReDoVs, and the data are depicted in Fig. 1.

### The prevalence of ReDoVs in nasopharyngeal samples

**Fig. 2:**
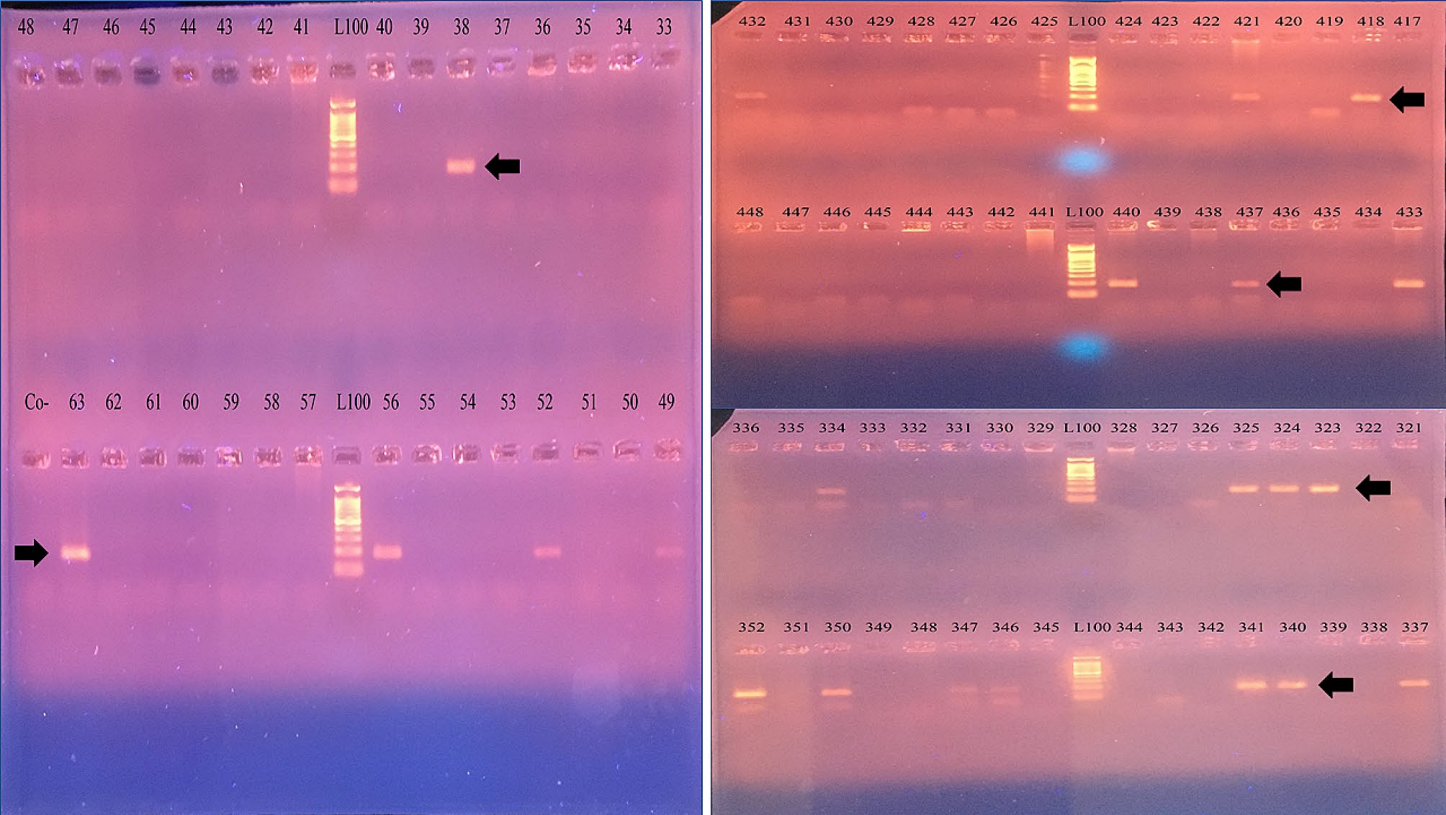
PCR amplification and gel electrophoresis of ReDoV genome. The gel electrophoresis demonstrates the presence of ReDoV DNA in the positive samples (Black arrows) with a size of 187 nt long. L100 represents the DNA marker (ladder) for size reference, while lane numbers show the PCR products of individual samples

**Fig. 3:**
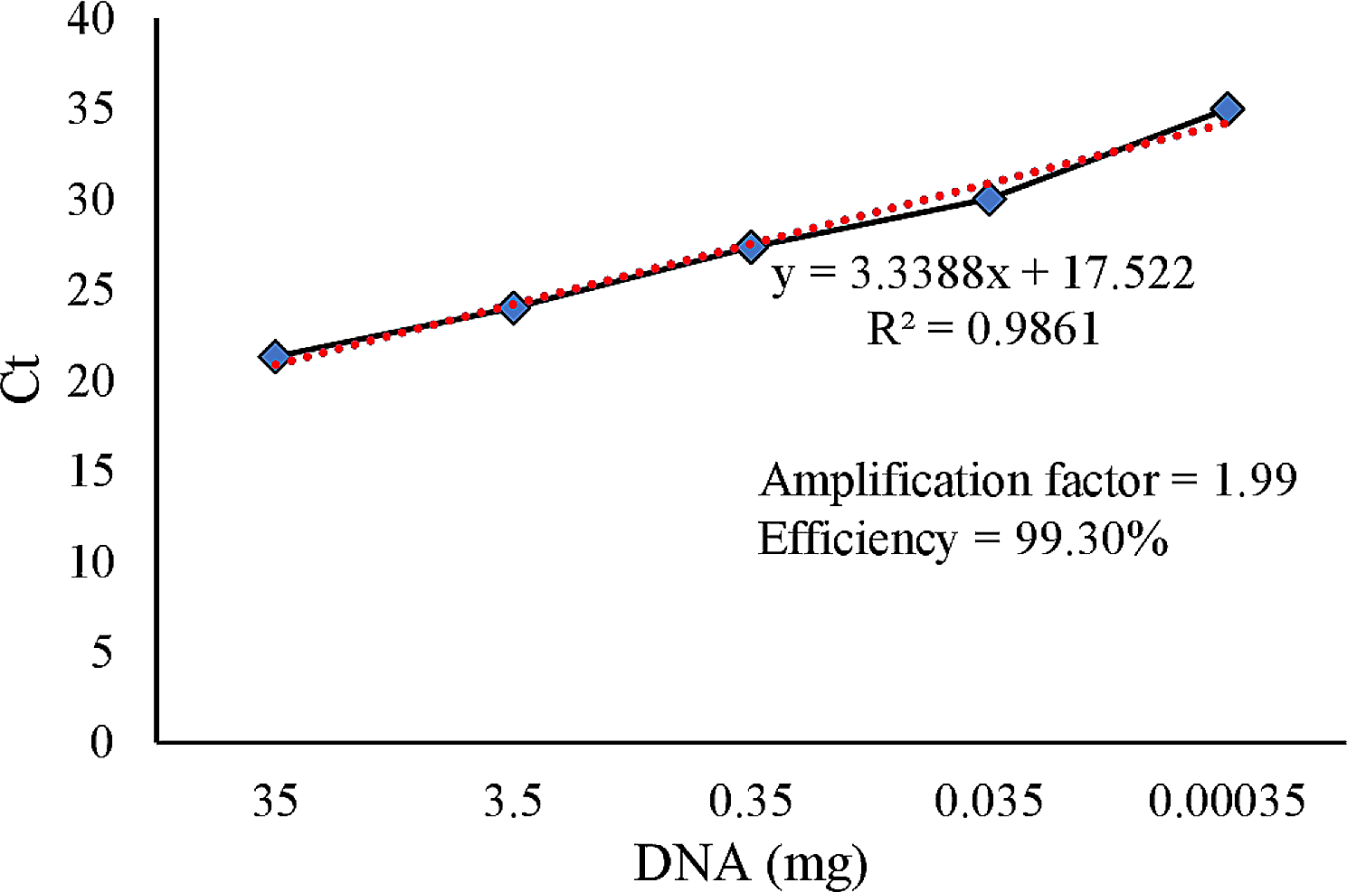
Calculated PCR efficiency with a rounded slope of 3.34. 350 ng viral DNA was also detected by this PCR method

A set of primers was designed to encompass a conserved region from nucleotides 1709 to 1895 (amplicon size of 187nt), covering 54 nt end of the C-terminal region of the Capsid coding region and the gap before starting the Rep coding sequence. Figure 2 displays the PCR amplification and gel electrophoresis results, showing specific bands corresponding to the amplified ReDoV DNA. Additionally, the efficiency of the PCR was 99.3% (Fig. 3).

ReDoV positivity was detected in 190/731 (25.99%) cases, with 29/190 (15.26%) exhibiting coinfection with SARS-CoV-2. The contingency table analysis was used to examine the relationship between ReDoVs presence and SARS-CoV-2 infection. The chi-square test of independence revealed no association between ReDoVs and SARS-CoV-2 infections (*p* > 0.05). Similarly, Fisher’s exact test showed no significant association between ReDoVs and SARS-CoV-2 (*p* > 0.05). These results indicate no statistical link between ReDoVs and SARS-CoV-2 infection among the studied individuals.

**Fig. 4:**
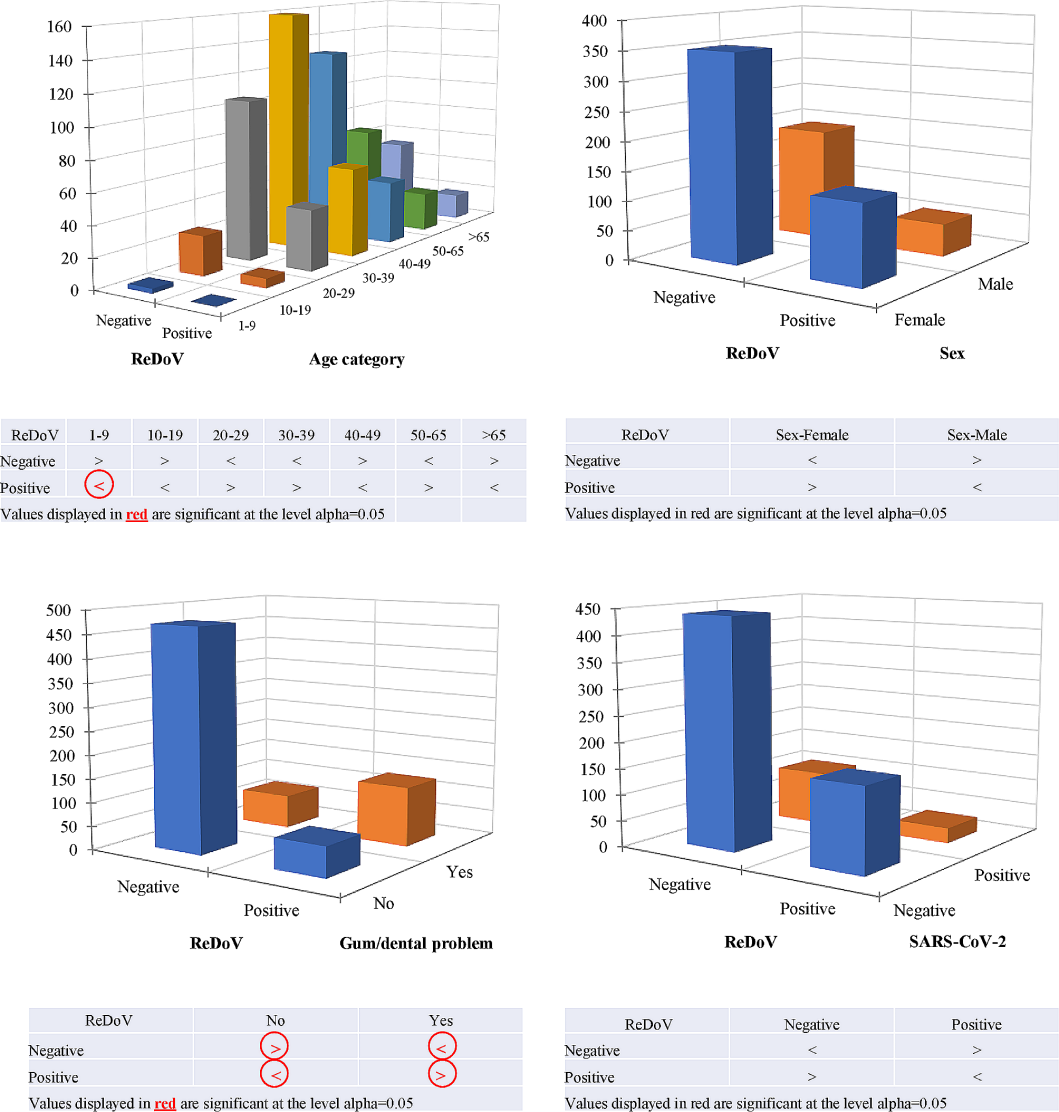
Association of ReDoVs genome prevalence in different age categories, sex, SARS-CoV-2 results, and gum or dental complications results. The Red arrows highlight the groups in which negative or positive results of ReDoV are significant based on Fisher’s exact test

Furthermore, the relationship between ReDoV infection and sex among individuals was investigated (Fig. 4). The positive results for ReDoVs among females and males were 135/731 (18.47%) and 55/731 (7.52%), respectively. No significant association was observed (*p* = 0.12), suggesting the variables’ independence. Furthermore, Fisher’s exact test for significance by cell also displayed no significant association between ReDoVs results and sex (*p* > 0.05).

Additionally, the relationship between ReDoVs and age categories was evaluated. Accordingly, the results showed that among those aged 1–9, 10–19, 20–29, 30–39, 40–49, 50–65, and > 65, there were 0, 6, 40, 59, 42, 26, and 17 individuals positive for ReDoVs within the corresponding age categories. The chi-square test of independence (*p* > 0.05) indicated that infection and age categories at ReDoVs are independent. Further analysis using Fisher’s exact test for significance by cell revealed no significant association between ReDoVs genome presence and age categories. However, Fisher’s exact test showed a non-random association in the absence of the ReDoVs genome in children aged 1–9. Nevertheless, due to the limited number of patients in this category, the virus’s prevalence in this age group needs further investigation. No significant associations were also found between other clinical variables.

### The association of ReDoVs with gum’s problems

The participants were questioned about any dental or gum inflammations or issues they had during their referral to the medical center. Surprisingly, the results revealed a highly significant relationship between infection with ReDoVs and dental/gum complications. The frequencies of negative and positive ReDoVs in the absence or presence of gum problems showed 126 individuals with gum problems tested positive for ReDoVs (*p* < 0.0001), indicating a link between ReDoVs presence and the reported gum problems. Additionally, Fisher’s exact test further corroborated the significant association, with *p* < 0.0001 for negative and positive ReDoVs results compared to the absence or presence of gum problems. Two sets of association coefficient tests were performed to measure further the strength of the association between the ReDoV infection and gum issues. According to the first set of statistics, Pearson’s Phi, Cramer’s V, and Tschuprow’s T all exhibit identical values of 0.5258, indicating a positive correlation between the variables. Cohen’s kappa also reflected a substantial agreement at 0.5256. Additionally, Yule’s Q and Yule’s Y coefficients show high values of 0.8575 and 0.5662, respectively, suggesting a strong positive association between ReDoVs and gum problems. The coefficients from the second set further support this relationship, with Goodman and Kruskal Gamma (0.8575 ± 0.0264), Kendall’s tau (0.5258 ± 0.0357), and Somers’ D of 0.5197 ± 0.0370 and 0.5319 ± 0.0372 for different orientations, all indicating a strong association. The calculated odds ratio (OR) was 13.0326 (95% CI:8.73–19.25), which signifies that individuals with gum problems are approximately 13 times more likely to test positive for ReDoVs than those without gum problems. Further information is provided in Table 2.

**Table 2:** Association coefficients of ReDoVs and gum/dental problems

Coefficient (1) ^¥^	Value	Coefficient (2) ^¥^	Value	Standard deviation	Lower bound 95%	Upper bound 95%
Pearson’s Phi	0.5258	**Goodman and Kruskal Gamma** ^**£**^	0.8575	0.0264	0.8057	0.9092
Contingency coefficient	0.4654	Kendall’s tau	0.5258	0.0357	0.4558	0.5958
Cramer’s V	0.5258	Stuart’s tau	0.4093	0.0331	0.3444	0.4742
Tschuprow’s T	0.5258	Somers’ D (R/C)	0.5197	0.0370	0.4473	0.5922
Goodman and Kruskal tau (R/C)	0.2765	Somers’ D (C/R)	0.5319	0.0372	0.4589	0.6049
Goodman and Kruskal tau (C/R)	0.2765	Theil’s U (R/C)	0.2251	0.0310	0.1644	0.2859
Cohen’s kappa	0.5256	Theil’s U (C/R)	0.2214	0.0306	0.1614	0.2813
**Yule’s Q** ^**£**^	0.8575	Theil’s U (Symmetric)	0.2232	0.0306	0.1632	0.2833
Yule’s Y	0.5662					

^**¥**^ The strength and direction of the relationship between ReDoVs and gum/dental problems

^**£**^ Goodman & Kruskal Gamma (0.8575 ± 0.264) and Yule’s Q coefficients represented the perfect positive relationship between ordinal variables ReDoVs positivity and reported gum/dental problems during the interview

## Discussion

A novel CRESS DNA virus from the *Redondoviridae* family was initially identified in lung transplant patients’ bronchoalveolar lavage (BAL) samples. ReDoVs, Vientovirus, and Brisavirus are proposed as the second most prevalent eukaryotic viruses in human respiratory samples [30]. CRESS DNA viruses are now ubiquitous and have been discovered on all continents, infecting various animals, including insects, birds, rodents, bats, chimps, and humans, as well as in diverse environmental and animal tissue samples. There is a limited understanding of ReDoV’s epidemiology, biological properties, and pathogenic potential.

Due to the lack of definitive evidence linking ReDoV to severe respiratory tract diseases, the present study aimed to investigate the association of ReDoV with human-associated viral diseases, particularly SARS-CoV-2 infections. SARS-CoV-2 has a significant impact on global public health [31–33], and human microbiota may affect its outcome [13]. This cross-sectional study comprised 731 biological samples from 486 females and 245 males referred for SARS-CoV-2 diagnostic testing. The study results provide data on the participants’ demographic traits and clinical aspects to explore possible links between ReDoV infection and SARS-CoV-2 and additional details gathered from interviews about dental issues, flu symptoms, and vaccinations for COVID-19 or Influenza A.

This study’s participants were mostly adults (20–49 years; 73.18%). As a result of the study, 17.92% of participants tested positive for SARS-CoV-2, and 97.13% received COVID-19 vaccination, mainly three times (45.69%) with Sinopharm being the most prevalent vaccine type (19.84%). A PCR assay was performed to evaluate the prevalence of ReDoVs in nasopharyngeal samples of the COVID-19-tested individuals. Different methods have been developed to investigate the genome of ReDoVs. Accordingly, an in-house developed two PCR protocols and rolling circle amplification (RCA) methods were used as a sensitive DNA amplification technique by Spezia et al. to detect ReDoVs in human respiratory, blood, stool, and liquor samples [30]. In that study, ReDoV DNA was detected in 4% of the population, with the highest prevalence (11%) in respiratory tract samples. Here, with our set of primers designed to amplify the minute amount of viral DNA with high efficiency, the ReDoV genome was found in 25.99% of nasopharyngeal samples, of which 15.26% exhibited coinfection with SARS-CoV-2. Nevertheless, there was no notable connection between ReDoVs and SARS-CoV-2 infections. This indicates that in this group, the presence of ReDoVs did not substantially associate with SARS-CoV-2 infection. A further study reports that Brazilian outpatients with mild COVID-19 have high ReDoV positivity (74.6%) in saliva samples of patients that did not significantly differ from the positive cases among the healthy control group (50.0%) [12]. Together with our findings of the ReDov genome in nasopharyngeal samples, saliva samples from the oral cavity might represent the ReDoVs as oral microflora or torque teno virus (TTV), a frequent member of the human virome [34]. As a further result of this study, ReDoV infection did not exhibit a sex-specific pattern within the studied population. As in this study, the number of female samples was larger than that of males; further investigation with sex-match is required to support this finding. However, since ReDoVs could exist as a part of the human virome, the sex distribution of the viral infection might not be the case. This finding paves the way for future studies on the possible mechanism of ReDoVs’ pathogenesis in periodontitis and other oral-associated complications.

Periodontal disease is linked to ReDoVs. Their frequency decreases following successful treatment, indicating a possible contribution to the advancement of the disease [5]. A significant relationship was observed between ReDoV infection and gum problems, as indicated by the high frequency of ReDoV-positive individuals reporting gum inflammation or issues during their referral to the medical centers. Various association coefficients supported this robust correlation, indicating a strong positive association between ReDoVs and gum problems. Furthermore, the calculated OR of 13.0326 (95% CI: 8.73–19.25) emphasized that individuals with gum problems were approximately 13 times more likely to test positive for ReDoVs than those without gum problems. A bidirectional association has been considered between oral health issues such as periodontitis and underlying inflammatory diseases like chronic kidney disease [16, 35], necessitating oral health maintenance programs [35]. Here, no association was found between underlying diseases and interview findings of gum/dental problems (data are not shown). Nevertheless, this indicates that there might be a relationship between ReDoV infection and inflammatory kidney diseases, which requires further study.

Unlike some other viruses, CRESS DNA viruses lack a universal gene for phylogenetic analysis. Therefore, a phylogeny tree was constructed based on the association between the ReDoVs and the isolation site of the virus in the human body. In this regard, three oral-, lung-, and human respiratory-associated ReDoVs were retrieved from NCBI’s taxonomy database. The generated tree demonstrated different assignment of the sequenced genome of detected ReDoVs in oral-associated Vientovirus (PP319427 and PP319428), lung-associated Vientovirus (PP3194424), and human respiratory-associated Brisavirus (PP319425 and PP319426). This phylogeny suggests that the nasopharyngeal samples are not very specific since the swabs could be contaminated with oral cavity or lung respiratory aspirates; therefore, detected ReDoVs can be assigned to different human association groups. Also, this helps to detect more numbers of viral genomes, suggesting an opportunity for uncovering novel members of *Redondoviridae.*

These results have significant implications for comprehending the dynamics of ReDoV infections, especially in public health. The strong connection with gum issues highlights the importance of viewing oral health as a possible risk factor or indicator for ReDoV infections. Moreover, the absence of a connection with age, gender, or SARS-CoV-2 infection emphasizes the distinct features of ReDoVs and the necessity for focused research on their epidemiology and clinical consequences. Recognizing the constraints of this research, such as its observational design and possible confounding factors, is crucial. The Fisher exact test revealed a notable lack of ReDoVs in children under ten. Nevertheless, more research is needed to better understand the frequency and effects of ReDoVs in specific age groups, such as children under ten, due to the limited number of reported cases in these categories. Furthermore, relying on self-reported data for particular variables like underlying diseases and symptoms in the study could lead to recall bias. Also, the study was not sex- and age-matched, and dental issues were not specified and characterized during the interview. Furthermore, a strong correlation has been reported between ReDoVs DNA and *E. gingivalis* DNA in the samples [16]. In this study, participants were questioned about their dental or gum issues, but the concurrent presence of *E. gingivalis* and ReDoVs was not examined, which will be included in our upcoming study. An additional limitation of the study is the inability to determine the specificity and sensitivity of the PCR test used for detecting ReDoV due to the unavailability of sufficient confirmed true positive and negative samples. Therefore, in our future research, we will validate the method to provide the PCR performance metrics.

## Conclusion

This cross-sectional study provided insights into the prevalence of ReDoVs in nasopharyngeal samples of people tested for COVID-19. In this regard, an efficient in-house PCR method observed between ReDoVd with a specific set of primers was designed to amplify a conserved region in the genome of ReDoVs, strengthening the chance of viral genome detection. Accordingly, the finding highlighted that 1/4 of the tested samples were positive for ReDoVs. No association was observed between ReDoV presence and SARS-CoV-2 infection. However, a significant link was observed between ReDoV infection and gum/dental problems with or without SARS-CoV-2 infection. This highlights the importance of oral health considerations in viral infections. Further research is warranted to expand our understanding of ReDoVs, their clinical implications, and potential preventive measures.

### Electronic supplementary material

Below is the link to the electronic supplementary material.

Supplementary Material 1

## Data Availability

The datasets generated and/or analyzed during the current study are available in the GenBank (https://www.ncbi.nlm.nih.gov/nucleotide/) repository under the accession numbers PP319424 to PP319438.
