# Supplementary material for: Dental complications as a potential indicator of Redondovirus infection: a cross-sectional study
Source: BMC Infect Dis. 2024 Jul 5;24:673. doi: 10.1186/s12879-024-09523-6 (PMC11225247; doi:10.1186/s12879-024-09523-6)
Supplement: Supplementary file 1 — Supplementary Material 1 [file 12879_2024_9523_MOESM1_ESM.pdf]

Questionnaire of a proposal entitled “Examining the presence of Redondovirus infection in the gingival samples of patients with apical or severe periodontitis and the gingival samples of healthy individuals referring to health centers related to Iran University of Medical Sciences“

Project Code: 26417

Ethical code of the study: IR.IUMS.FMD.REC.1402.351

Note 1: The implementation of this study is subject to ethical considerations.

Note 2: The questionnaire only pertains to the answers given when individuals are referred for COVID-19 tests.

Individual code:

Gender:

Male ☐ Female ☐

---

1. Has the person received the COVID-19 vaccine?

Yes ☐ If yes, how many doses have they received? … No ☐

* Please mention the vaccine(s) brand name:

2. Has the person received the influenza vaccine this year?

Yes ☐ If yes, how many doses have they received? … No ☐

3. Does the person have any specific diseases? If yes, please explain.

Yes ☐ If yes, please **name them**. … No ☐

4. Does the person receive any specific medication? (If yes, what medication does the person receive?)

Yes ☐ If yes, what medication does the person receive? … No ☐

5. Does the person have any mouth and teeth issues?

Yes ☐ No ☐

6. Does the individual exhibit any of the following symptoms? (Check all that apply)

Fever ☐
Chills ☐
Dry cough ☐
Chest pain ☐
Runny nose ☐
Vomiting ☐
Diarrhea ☐
Stomach bleeding ☐

Coldness ☐
Tremors ☐
Back pain ☐
Joint pain ☐
Muscle pain ☐
Headache ☐
Weakness ☐
Loss of taste ☐
Loss of smell ☐

7. Did the person feel they had a high fever at the preferred time?

Yes ☐ No ☐

If you have any questions or concerns about this study, please get in touch with the investigator(s), at the following contact addresses:

+98 935467 4593

Alirezaa2s@gmail.com
